# An Insight into the Mechanism of DNA Cleavage by DNA Endonuclease from the Hyperthermophilic Archaeon *Pyrococcus furiosus*

**DOI:** 10.3390/ijms25168897

**Published:** 2024-08-15

**Authors:** Anastasiia T. Davletgildeeva, Aleksandra A. Kuznetsova, Alexander A. Ishchenko, Murat Saparbaev, Nikita A. Kuznetsov

**Affiliations:** 1 Institute of Chemical Biology and Fundamental Medicine, Siberian Branch of Russian Academy of Sciences, Novosibirsk 630090, Russia; davleta94@gmail.com (A.T.D.); sandra-k@niboch.nsc.ru (A.A.K.); 2 Group «Mechanisms of DNA Repair and Carcinogenesis», CNRS UMR9019, Gustave Roussy Cancer Campus, Université Paris-Saclay, F-94805 Villejuif CEDEX, France; alexander.ishchenko@gustaveroussy.fr (A.A.I.); murat.saparbaev@gustaveroussy.fr (M.S.); 3 Department of Natural Sciences, Novosibirsk State University, Novosibirsk 630090, Russia

**Keywords:** DNA repair, apurinic/apyrimidinic endonuclease, abasic site, damaged nucleotide, endonuclease activity, exonuclease activity, pre-steady-state enzyme kinetics

## Abstract

Hyperthermophilic archaea such as *Pyrococcus furiosus* survive under very aggressive environmental conditions by occupying niches inaccessible to representatives of other domains of life. The ability to survive such severe living conditions must be ensured by extraordinarily efficient mechanisms of DNA processing, including repair. Therefore, in this study, we compared kinetics of conformational changes of DNA Endonuclease Q from *P. furiosus* during its interaction with various DNA substrates containing an analog of an apurinic/apyrimidinic site (F-site), hypoxanthine, uracil, 5,6-dihydrouracil, the α-anomer of adenosine, or 1,*N*^6^-ethenoadenosine. Our examination of DNA cleavage activity and fluorescence time courses characterizing conformational changes of the dye-labeled DNA substrates during the interaction with EndoQ revealed that the enzyme induces multiple conformational changes of DNA in the course of binding. Moreover, the obtained data suggested that the formation of the enzyme–substrate complex can proceed through dissimilar kinetic pathways, resulting in different types of DNA conformational changes, which probably allow the enzyme to perform its biological function at an extreme temperature.

## 1. Introduction

The native structure of DNA of all living organisms from bacteria to mammals is constantly subjected to alterations due to the impact of numerous endogenous and exogenous factors, such as spontaneous hydrolysis, reactive oxygen species, and other chemically active metabolites, as well as misincorporation events during replication [1,2,3].

Among the four DNA bases, cytosine is the most sensitive to high-temperature-induced degradation, with spontaneous hydrolytic deamination of cytosine occurring at neutral pH and leading to the formation of uracil (U) in DNA [4,5]. In addition, hydrolytic deamination of cytosine can be caused by specific cytidine deaminases [6]. Another important source of U in DNA is the misincorporation of dUMP instead of dTMP by DNA polymerases [7]. The emergence of U in DNA is highly mutagenic because it tends to pair with adenine, leading to the GC→AT substitution [4]. Moreover, the removal of U by specific uracil DNA glycosylases (UDGs) during base excision repair (BER) gives rise to a highly mutagenic site (apurinic/apyrimidinic (AP) site) as an intermediate [8,9,10]. The action of specific DNA glycosylases is not the only source of AP sites in DNA, because such sites can result from spontaneous hydrolysis of an *N*-glycosidic bond due to its destabilization by various base lesions [11]. Hydrolytic deamination of adenine and guanine occurs in DNA under normal physiological conditions, thereby leading to the formation of hypoxanthine (Hx) and xanthine (X), respectively, in DNA [4,12], with the rate of spontaneous conversion of adenine to Hx being 2% of the rate of the conversion of cytosine to uracil [4]. Hx is a potentially mutagenic DNA lesion because it can pair not only with thymine but also with cytosine and adenine, and the introduction of Hx has been shown to induce the AT→GC substitution [13,14]. Oxidation of cytosine via exposure to reactive oxygen species (arising in the cell during oxidative reactions resulting from either endogenous cellular processes or exposure to exogenous factors [15]) generates such premutagenic lesions as 5-hydroxycytosine (5hC), 5-hydroxyuracil (5hU), and 5,6-dihydrouracil (DHU) [10].

Given that all the lesions mentioned above are potentially mutagenic and arise in DNA constantly, living organisms have developed various DNA repair systems in the course of evolution to preserve DNA integrity. One of the most important pathways is BER. It implements the removal of U from DNA by UDGs. The resultant AP site is cleaved by AP endonuclease, and native DNA structure is restored by sequential actions of DNA polymerase, ligase, and some coordinating proteins, such as XRCC1 and PCNA, involved in the BER process [10,16,17]. Hx is also removed by a BER enzyme called alkyladenine DNA glycosylase (AAG) [18]. Additionally, endonuclease Endo V from *Escherichia coli* is capable of cleaving the second phosphodiester bond on the 3′ side of the deaminated bases, including Hx and U, during alternative excisional repair [19,20,21]. Although it is widely accepted that the main cellular function of AP endonucleases is the cleavage of DNA on the 5′ side of an AP site, it has been found [22,23] that prokaryotic AP endonuclease Nfo from *E. coli* hydrolyzes the phosphodiester bond in DNA on the 5′ side of some oxidatively damaged bases via a pathway independent of DNA glycosylases, which is called nucleotide incision repair (NIR). Later, it was demonstrated that some AP endonucleases can recognize as substrates a variety of damaged bases, for example, 2′-deoxyuridine [24], oxidatively damaged pyrimidines [25], bulky photoproducts [26], benzene-derived DNA adducts [27], α-anomers of 2′-deoxynucleosides [23], and etheno-derivatives of DNA bases [28,29].

Most pro- and eukaryotic AP endonucleases belong to one of the two well-known families: Xth (or ExoIII) and Nfo (or EndoIV) [30,31,32,33,34]. Members of both families combine BER and NIR functions and are able to hydrolyze a DNA phosphodiester bond on the 5′ side of an AP site and various damaged nucleotides [22,35,36,37]. Although the molecular mechanism underlying the broad substrate specificity of AP endonucleases is still debated [38,39,40], members of Xth and Nfo families have common features of interaction with damaged DNA. Analysis of X-ray data clearly indicates that in all known enzyme–substrate complexes of AP endonucleases [33,41,42,43], the DNA backbone is bent, the damaged nucleotide is placed in the active-site pocket, and a few amino acid residues of the enzyme are wedged into the formed void in the DNA duplex. Analyses of substrate specificity of human APE1 from the Xth family by pulsed electron–electron double resonance (PELDOR) spectroscopy [38], pre–steady-state kinetics [44,45,46], and molecular dynamics (MD) simulations [40] have unexpectedly revealed that the enzyme does not form any damage-specific contacts in the active-site pocket. These data suggest that the ability of a damaged nucleotide to flip out from DNA and to penetrate the enzyme pocket in response to an enzyme-induced DNA distortion and bending is a key contributing factor of substrate specificity.

Moreover, researchers have identified [47,48,49,50] a novel endonuclease class of enzymes (from archaea and some bacteria) designated as Endonuclease Q (EndoQ), which possesses both AP site specificity and unique specificity for deaminated nucleobases such as U and Hx in DNA. Later, it was revealed that EndoQ from *Pyrococcus furiosus* also has activity toward 5hU and DHU [51]. The group of scientists who first isolated EndoQ has also proposed a possible role of this enzyme in the NIR pathway [48]. Moreover, it seems that EndoQ plays an important part in U removal in some organisms, thereby complementing the action of AP endonuclease and UDG and sometimes compensating for the absence of one of these enzymes [52]. Of note, EndoQ homologs are widespread among representatives of the archaeal domain and are present in some bacteria; however, no homologous enzymes have been found among eukaryotes yet [48].

Among all known AP endonucleases, EndoQ is the only enzyme that has been crystalized in a catalytic complex with DNA containing damaged bases [50]. It has been shown [50] that EndoQ-bound DNA is also sharply bent by ∼40° away from the catalytic domain. The damaged nucleotide is at the apex of the kink, is rotated out of the double helix, and is buried in a deep pocket formed between the catalytic and Zn-binding domains. It has been reported that the enzyme induces the base-flipping via DNA backbone distortion without any protein side chain “wedging.” Moreover, by modeling undamaged bases in the U/Hx-binding pocket of EndoQ, some authors have found that the selectivity of EndoQ is based on the steric ability of the base to fit into the enzyme pocket. Consequently, it could be concluded that the substrate specificity and the nature of the catalytic cleavage of damaged DNA by EndoQ are very similar to those of AP endonucleases from the Xth and Nfo families. Considering the fact that the novel enzyme possesses no structural similarity to any known AP endonucleases [53], it is possible that EndoQ represents a new class of multifunctional AP endonucleases, and its potential role in the BER and NIR pathways of archaea is of interest to researchers.

Therefore, the main purpose of our study was to compare kinetics of conformational changes of EndoQ from *P. furiosus* during its interaction with various damaged DNA substrates, including DNA duplexes containing an F-site (an analog of an AP site), Hx, or U. To better understand the specificity of substrate recognition by the enzyme, we studied the kinetics using different nucleotides opposite the damaged base. Moreover, to investigate the possible function of EndoQ in NIR, we examined substrate specificity of the enzyme toward other typical NIR substrates, such as the α-anomer of adenosine (αA) and 1,*N*^6^-ethenoadenosine (εA).

## 2. Results

### 2.1. Effects of Metal Ions

**Figure 1:**
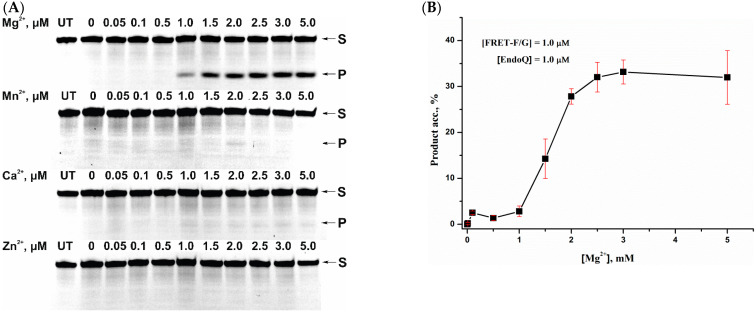
(**A**) Analysis of DNA cleavage by EndoQ during an interaction with substrate F/G in the presence of different concentrations of divalent metal ions, conducted in denaturing 20% (*w*/*v*) PAGE. S and P are substrate and product, respectively. UT stands for the substrate untreated with the enzyme. (**B**) Effects of the Mg^2+^ concentration on the efficiency of substrate F/G cleavage by EndoQ. [EndoQ] = [substrate F/G] = 1.0 μM. T = 40 °C, reaction time = 3 min.

The effects of reaction conditions such as pH, monovalent ion concentration, the nature of the divalent cation, and temperature on the activity of EndoQ were reported previously by Ishino et al. [47]. It was shown by them that the relative activity of EndoQ toward Hx-containing DNA is maximal at 75 °C in a buffer containing 1.0 mM MgCl_2_. A comparison of the related DNA cleavage activity revealed a dependence of the activity on the nature of divalent cations in the reaction buffer in the following order: Mn^2+^ > Mg^2+^ > Ca^2+^ >> Zn^2+^ ≈ EDTA. It has been reported [50,53] that EndoQ possesses three Zn^2+^ cations in its structure, but the roles of these divalent cation in EndoQ activities are unknown. At the same time, it is known that the DNA binding of EndoQ is dependent on Mg^2+^ [48]. Considering the fact that the effect of divalent cations was analyzed in Ref. [47] only at a single concentration of metal ions, we performed a DNA cleavage activity assay of EndoQ toward DNA containing an abasic site at various concentrations of metal ions at 40 °C (Figure 1A).

Given that Mg^2+^ apparently is not fixed in the structure of the enzyme, EndoQ was preincubated with 1.0 mM EDTA to remove any divalent cations from the active site of the enzyme in order to make the enzyme catalytically inactive. This metal-free EndoQ was then supplemented with various concentrations of metals. It was found that only Mg^2+^ ions could reactivate EndoQ, whereas the enzyme had extremely low activity toward substrate F/G at 40 °C in the presence of any surrogate metals (Mn^2+^, Ca^2+^, or Zn^2+^) for up to 30 min of the reaction time (Figure 1A).

These data indicated that EndoQ has maximal cleavage efficiency toward substrate F/G in the presence of Mg^2+^ at concentrations of 2 mM and higher (above 1 mM EDTA) (Figure 1B). Moreover, no other divalent metal cation under study was able to restore the enzymatic activity. Based on these findings, further enzyme assays were performed in a buffer supplemented with 2 mM MgCl_2_.

### 2.2. Base-Pairing Effects

Siraishi and Iwai have shown that the activity of EndoQ toward DNA substrates containing such lesions as U, 5hU, and Hx depends on the stability of the base pair of the damaged base [51]. They suggested that the cleavage efficiency of EndoQ negatively correlates with the base pair stability. To investigate how these effects influence the profile of FRET signal changes and to additionally determine the influence of the base in the complementary strand in case of an abasic site, base-pairing effects were investigated using PAGE and stopped-flow experiments.

**Figure 2:**
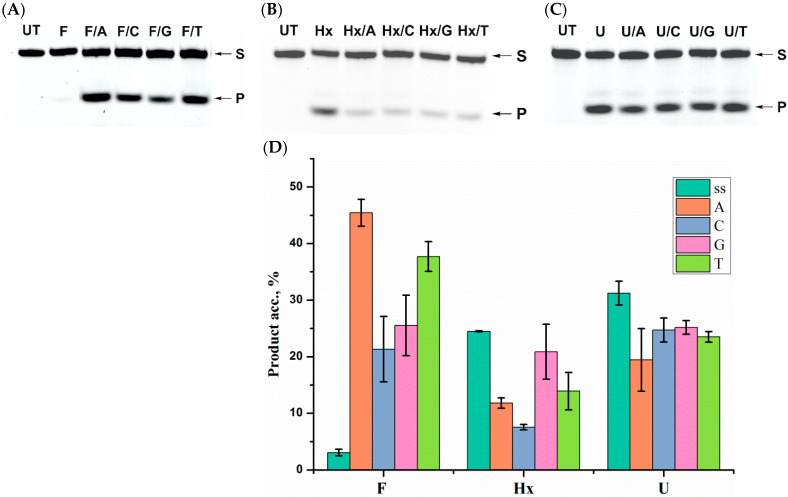
Analysis of DNA cleavage by EndoQ during an interaction with the F-substrate (**A**), Hx-substrate (**B**), or U-substrate (**C**), conducted in denaturing 20% (*w*/*v*) PAGE. The damaged bases or the F-site were placed opposite A, C, G, or T in dsDNA or remained sole (ssDNA). S and P are substrate and product, respectively. UT stands for the substrate untreated with the enzyme. (**D**) A comparison of cleavage efficacy of model DNA substrates by EndoQ depending on the opposite nucleotide or its absence. [Enzyme] = [DNA substrate] = 1.0 μM. T = 40 °C, reaction time = 3 min.

The comparison of cleavage efficiency of EndoQ toward the DNA substrates containing F, Hx, or U opposite A, C, G, or T in 17-bp double-stranded DNA (dsDNA) or ssDNA was conducted by PAGE. The choice of the model DNA substrates made it impossible to investigate the activity of EndoQ toward ssDNA containing various lesions using the stopped-flow method because the BHQ1 quencher (essential for FRET signal detection) is at the 5′ end of the complementary strand of the duplex and not at the 3′ end of the cleavable strand containing 5′-FAM. Cleavage efficiency of EndoQ observed in the PAGE experiments could be ranked as follows: F/A > F/T > F/C > F/G > F, Hx > Hx/G > Hx/T > Hx/A > Hx/C, and U > U/G ≈ U/C > U/T > U/A (Figure 2). Unexpectedly, Hx and U in ssDNA turned out to be the most efficiently cleavable substrates for EndoQ compared to all four dsDNA substrates, whereas F in ssDNA proved to be a very unsuitable substrate, consistently with data obtained earlier [48,51]. The level of the product accumulation was the lowest for Hx/C and U/A among all four substrates, and these two are the most stable pairs for these lesions [51,54]. It is worth noting that, taking experimental error into account, the overall difference in cleavage efficiency of the enzyme toward lesions among different contexts was quite moderate. The levels of the product accumulation were especially similar in the case of the U-containing DNA. It is possible that the discrepancies between our data and the earlier results are due to some differences in the experimental conditions or to the influence of the context of DNA substrates. When different lesions were compared, the most effectively cleaved damage was F; U and Hx were somewhat less suitable and were similar to each other in this regard. Nonetheless, U was cleaved more efficiently than Hx.

**Figure 3:**
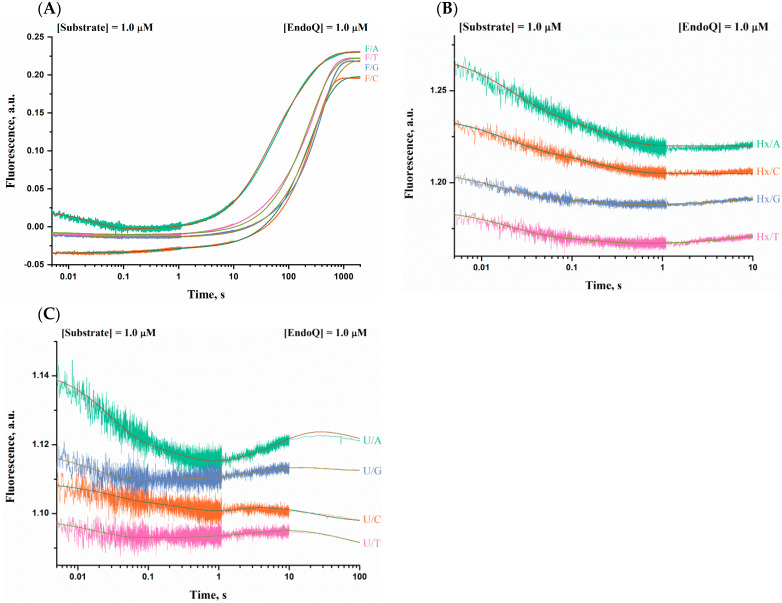
Changes in the FRET signal during the interaction of EndoQ with the F- (**A**), Hx- (**B**), or U-containing (**C**) DNA substrate. Damaged bases were paired with A, C, G, or T. Individual traces were fitted to several exponential equations, with the number of exponents being 2, 3, or 4.

To estimate the impact of base pairing on kinetic features of the interaction of EndoQ with damaged DNA substrates, 1 µM DNA substrate containing F, Hx, or U opposite A, C, G, or T was rapidly mixed with 1 µM EndoQ in the stopped-flow apparatus, and fluorescence was monitored with time. Different lesions gave quite distinct types of kinetic traces, although profiles of the curves were more or less similar among different nucleotides opposite the same lesion (Figure 3).

For the F-containing DNA substrate, the kinetic curves contained a small initial decrease in the signal (except for F/C), which was most pronounced for F/A. This decrease most likely reflects the FAM fluorophore and the BHQ1 quencher coming closer together, while the DNA substrate was being bent in a complex with EndoQ [50]. The curve of substrate F/C lacked the initial decrease stage but contained an additional long phase of the increase in the FRET signal with small amplitude, undetectable in the three other traces. The apparent small amplitude of the first stage was due to the very high amplitude of the final stage of the kinetic curve, most likely characterizing the cleavage step of the kinetic mechanism. This supposition is supported by results obtained in our laboratory previously for APE1 endonucleases [55] and by the fact that the F-containing DNA is cleaved during the periods in which the growth phase occurred (Figure 2). Notably, all four substrates’ curves reached a plateau within 1300 s.

The traces characterizing the interaction of EndoQ with substrates F/C, F/G, and F/T matched the double-exponential equation well, yielding the observed rate constants listed in Table 1. The double-exponential equation was a poor fit for the kinetic trace of substrate F/A; accordingly, in this case, the equation with three exponents was employed. The first observed rate constant *k*_1_ for kinetic traces of F/A, F/G, and F/T corresponds to the initial decrease in fluorescence, being the fastest for the F/T DNA substrate (53 s^−1^) and nearly the same between F/A and F/G (33 and 34 s^−1^, respectively). This rate constant may describe one of the stages of the initial substrate–enzyme complex formation. The exponential fitting of the kinetic trace for DNA substrate F/C did not give the rate constant that could be attributed to this stage of interaction between EndoQ and F-containing DNA. Instead, it yielded an observed rate constant *k*_2_ (2.15 s^−1^) corresponding to the long phase of the increase in the FRET signal, absent in any other curve in this series. Of note, the exponential fitting of the kinetic trace characterizing the interaction of the EndoQ with substrate F/A also yielded a unique observed rate constant *k*_3_ (0.030 s^−1^), matching the part of the curve where the signal started to grow. This makes the increasing portion biphasic, indicating that the increase in the FRET signal might be more complex, reflecting not only the catalytic cleavage process but also additional conformational adjustment of the DNA substrate in the active site of the enzyme. Finally, the differences in the last observed rate constant *k*_4_ fully correspond to the visual difference in the reaction rates, with *k*_4_ for substrate F/A being the fastest (0.005 s^−1^) and *k*_4_ for substrate F/G being the slowest (0.003 s^−1^). This observed rate constant most likely represents observed *k_cat_*. It should be pointed out that the order of cleavage efficiency toward the F-containing dsDNA was consistent between the PAGE and stopped-flow experiments, with the difference in *k*_4_ values being less than 0.002 s^−1^. Furthermore, *k*_4_ for F/A obtained in our experiments was ~5 times smaller than the observed *k_cat_* obtained earlier by Shiraishi and Iwai [51]. A possible reason is that due to technical limitations of the stopped-flow method, the reaction temperature chosen in our experiments (40° C) was 20° C lower than the one used by Shiraishi and Iwai, whereas the optimum for EndoQ was reached at an even higher temperature (75 °C) [47]. Additionally, the efficiency of the enzyme could have been affected by the difference in the DNA substrates’ lengths (17 nt in our experiments and 30 nt in Shiraishi and Iwai’s work).

The interaction of EndoQ with Hx- and U-containing DNA substrates yielded kinetic curve profiles that were quite distant from the ones for the F-containing DNA substrate. It must be noted that all of the kinetic curves included the final amplitude increased the phase but none reached a plateau, thus making it impossible to characterize the catalytic step of the reaction between EndoQ and the damaged DNA substrates. This is probably because Hx and U turned out to be less suitable substrates compared to the F-containing DNA.

The fluorescence traces characterizing the interaction between the EndoQ enzyme and the DNA substrate containing Hx opposite A, C, G, or T are presented in Figure 3B. The initial two-phase decrease in the FRET signal was followed by a slow increase with small amplitude, followed by a fast intensive increase in fluorescence going up to 4000 s.

The traces describing the interaction of EndoQ with substrate Hx/A or Hx/C were fitted to a double-exponential equation. The fluorescent curves for DNA substrates Hx/G and Hx/T matched the equation with three exponents well. Fitting of the kinetic curves up to 10 s yielded the values of observed rate constants listed in Table 2. The first observed rate constant *k*_1_ was of the same order of magnitude as in case of the F-containing DNA substrates, suggesting that it reflects the same aspects of the binding by EndoQ. *k*_1_ values for Hx/A, Hx/C, and Hx/T were very similar, and *k*_1_ of Hx/G was the fastest: ~70 s^−1^. The second observed rate constant *k*_2_ corresponds to the second phase of the initial decrease in the FRET signal and manifested exactly the same distribution of values, being ~5 s^−1^ in the case of Hx/A, Hx/C, and Hx/T and 8 s^−1^ for Hx/G. The additional increase phase, visually detectable for the Hx/T substrate, yielded an additional observed rate constant, *k*_3_. Of note, this phase was also present in the trace characterizing conformational changes of the Hx/G DNA substrate. Unfortunately, the slow cleavage process did not allow us to determine the observed catalytic rate constants in the case of Hx-containing DNAs.

The fluorescent traces for the interaction of EndoQ with the DNA substrate containing U opposite A, C, G, or T (Figure 3C) had a profile even more complicated than that of the Hx-containing DNA, with the changes in the signal being most intensive for DNA substrate U/A. The initial stage of the two-phase decrease in the FRET signal was followed by an increase stage starting after 1 s. This phase had smaller amplitude and was followed by another decrease stage with low magnitude of the fluorescence changes. The fluorescent traces characterizing conformational changes of substrates U/C, U/G, and U/T during their interaction with EndoQ had a similar profile but with smaller amplitudes of the changes in the signal. Moreover, the traces of U/G and U/T had a one-stage fast signal decrease mostly finished before time point 0.1 s.

The kinetic curves characterizing the interaction of EndoQ with U-containing DNA fitted an equation with three exponents well in the case of U/G and U/T and an equation with four exponents well in the case of U/A and U/C. The fitting of the kinetic curves up to 100 s yielded the observed rate constants listed in Table 3 The absence of the final plateau phase after the growth of the FRET signal (reflecting the catalytic stage of the interaction up to time point 4000 s) made it impossible to process the curves across the entire time range registered. The first observed rate constant *k*_1_ had the same value between substrates U/A and U/G (36 s^−1^) and was almost twice as high for U/G and U/T (60 and 55 s^−1^, respectively). This rate constant—reflecting the initial decrease stage of the traces—was of the same order of magnitude as that of F- and Hx-containing DNA. This similarity among all three lesions opposite different nucleotides suggests that the processes of the initial binding by EndoQ were almost the same among these substrates. The fitting to the exponential equation yielded the second observed rate constant *k*_2_, corresponding to the second phase of the initial decrease in the FRET signal only in the case of substrates U/A and U/C. Its values were 3.5 and 2.0 s^−1^, respectively. This was a bit smaller compared to Hx-containing DNA substrates but still of the same order of magnitude. Notably, this stage was well pronounced for all types of the opposite base for Hx but was absent for U/G and U/T. The subsequent increase phase is described by observed rate constant *k*_3_. It was the fastest for U/C (0.9 s^−1^) and the slowest for U/A (0.13 s^−1^) and was the same between U/G and U/T. Observed rate constant *k*_4_—matching the next decrease stage of the FRET signal with small intensity—had the smallest value for U/A and was the fastest in the case of U/C (0.005 and 0.038 s^−1^, respectively).

### 2.3. Interaction with DNA Containing F-Site, Hx, or U

**Figure 4:**
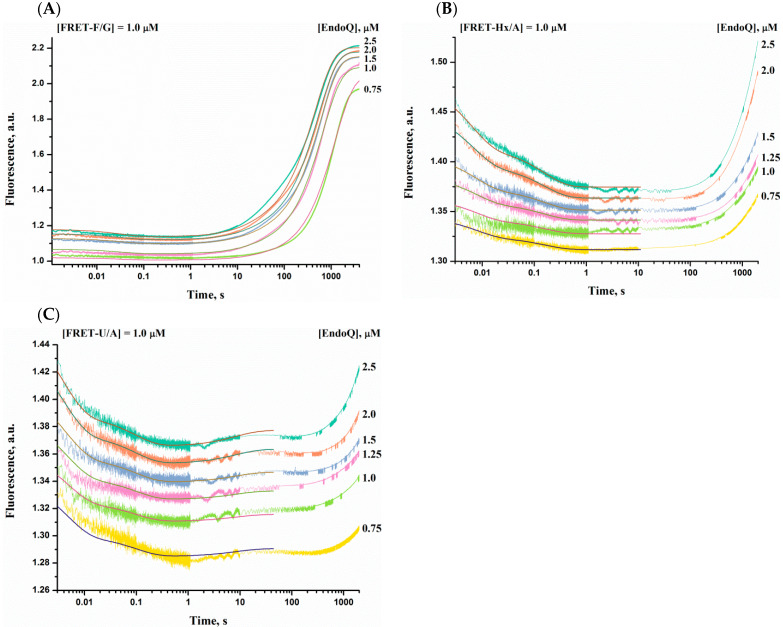
Changes in the FRET signal during the interaction of EndoQ with DNA substrate F/G (**A**), Hx/A (**B**), or U/A (**C**). [DNA] = 1.0 µM, concentrations of the enzyme are shown to the right of the panel. The traces were fitted using nonlinear least-squares regression analysis.

To elucidate the kinetic mechanism of interaction between EndoQ and F-, Hx-, or U-containing DNA substrates, a fixed concentration of the DNA substrate (F/G, Hx/A, or U/A) was rapidly mixed with various concentrations of the enzyme by the stopped-flow apparatus, and fluorescence was recorded for some time (Figure 4). The F/G substrate was chosen because this substrate gave fluorescent traces reaching a plateau within the period of the recording; this property was necessary for the analysis of the catalytic activity of the enzyme. Hx/A and U/A were chosen specifically because the fluorescent curves characterizing the interaction of EndoQ with these substrates manifested the most pronounced changes in the FRET signal. All three DNA substrates yielded the patterns of the kinetic traces identical to those obtained in the experiments, elucidating the base-pairing effects discussed above. It must be pointed out that in the case of substrate F/G, the additional phase of the growth of the signal similar to that of F/A occurred at higher concentrations of the enzyme (Figure 4A), suggesting that the process this phase matches might be common among all the F-containing DNA substrates under study.

**Scheme 1:**
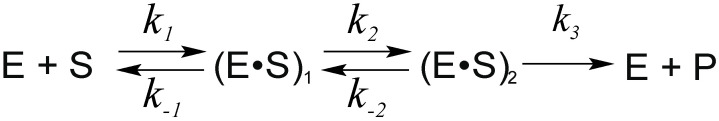
Interaction of the EndoQ enzyme with DNA substrate F/G.

The fluorescent traces describing conformational changes of dye-labeled substrate F/G during the interaction with EndoQ were fitted in DynaFit to a three-step mechanism (Scheme 1) to obtain rate and equilibrium constants (Table 4). The first and second stages of the minimal kinetic mechanism most likely correspond to the processes of substrate binding and formation of catalytically active complex (E·S)_2_. The next, irreversible stage reflects combined processes of the catalytic reaction and dissociation of the enzyme–product complex. It is worth mentioning that the *k_cat_* obtained for F/G in this series of experiments was of the same order of magnitude as the one determined for F/G by exponential fitting of the single curve (0.008 and 0.0031 s^−1^, respectively). The 2.5-fold difference might have been due to the different features of the experiments.

**Scheme 2:**
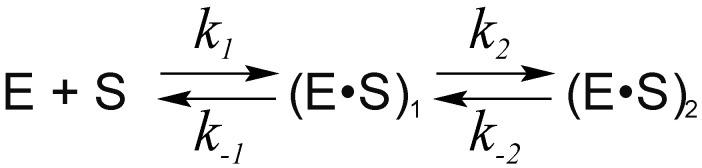
The binding of the Hx/A DNA substrate by EndoQ.

**Scheme 3:**
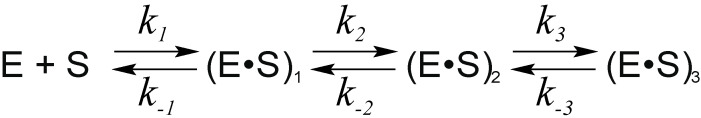
The binding of the U/A DNA substrate by EndoQ.

Next, the kinetic curves for the interaction of EndoQ with DNA substrate Hx/A or U/A were fitted to calculate rate and equilibrium constants (Table 4). The minimal kinetic mechanism contained two reversible stages in the case of Hx/A (Scheme 2) and three reversible stages for U/A (Scheme 3). The first stage of the mechanism reflects the formation of the initial enzyme–substrate complex (E·S)_1_ and is characterized by forward and reverse rate constants *k*_1_ and *k*_−1_ and by equilibrium constant *K*_1_, calculated as the ratio of *k*_1_ to *k*_−1_ (*K*_1_ = *k*_1_/*k*_−1_). The forward and reverse rate constants *k*_2_ and *k*_−2_ and the equilibrium constant *K*_2_ describe subsequent transformation of the (E·S)_1_ complex into (E·S)_2_, which could be the catalytically active form of the enzyme–substrate complex or one of its preceding forms. The first two stages were present in both kinetic mechanisms for Hx/A and U/A and most likely correspond to the same processes acting on DNA substrates during the binding by EndoQ. These stages were accompanied by a FRET signal decrease reflecting the FAM fluorophore and the BHQ1 quencher coming closer together when DNA bended in a complex with EndoQ. There were also two stages matching the binding process and further transformation of the substrate–enzyme complex in the kinetic mechanism underlying the interaction of F/G with EndoQ, but due to the differences in the fluorescent traces’ profiles, it is hard to be sure about the similarity of these processes between F/G, Hx/A, and U/A.

Furthermore, unlike any other damaged DNA substrate, U/A showed a third stage of increase in the FRET signal, taking place from second 1 to second 40 and leading to the formation of substrate–enzyme complex (E·S)_3_. Considering the time interval and the low intensity of the changes, this stage was hardly connected to the catalytic process and therefore must reflect some processes occurring to the bound DNA in complex with the enzyme. The formation of the initial enzyme–substrate complex (E·S)_1_ was less effective for Hx/A than for U/A owing to forward-reaction constant *k*_1_ being relatively slow (17 × 10^6^ M^−1^s^−1^). This caused a decrease in reaction constant *K*_1_ characterizing the stability of the (E·S)_1_ complex. It is worth noting that *K*_1_ was less than that for all three lesions, indicating that the initial substrate–enzyme complex might be quite unstable itself.

The formation of the second complex (E·S)_2_ proceeded approximately five times faster for U/A than Hx/A, although the overall stability of the complex was low for both substrates. Anyhow, the stability of the (E·S)_2_ complex in the case of the F/G DNA substrate was quite high, with a *K*_2_ of 5. The nature of this complex most likely differs between F/G, Hx/A, and U/A, thus making it inadequate to compare the kinetic parameters of this complex’s formation.

It might seem unusual that in the case of Hx/A and U/A we detected the emergence of two unstable complexes of the enzyme with the DNA substrate, but the catalytic process still occurred quite effectively according to the PAGE experiments. This peculiarity could be explained by the specifics of the method used. It is known that with U- and Hx-containing DNA, EndoQ forms complexes detectable by electrophoretic mobility shift assays [51]. Nonetheless, the complex visualized in the gel and resulting in the cleavage could form after the general bending of the DNA substrate, and its formation thus would not lead to any detectable changes in the FRET signal.

### 2.4. Interaction with DNA Containing Other Damaged Bases

**Figure 5:**
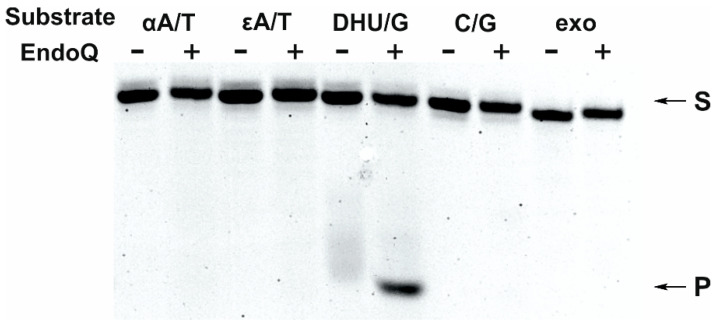
Analysis of EndoQ activity toward αA/T, εA/T, DHU/G, exo-, or undamaged (C/G) DNA substrates, conducted in denaturing 20% (*w*/*v*) PAGE. [Enzyme] = [DNA substrate] = 1.0 μM. T = 40 °C, reaction time = 30 min. S and P are substrate and product, respectively.

To investigate the substrate specificity range of EndoQ toward classic NIR substrates, namely, αA, εA, and DHU, cleavage efficiency of the enzyme toward the DNA substrates containing these lesions was analyzed by PAGE (Figure 5). EndoQ was able to cleave the DNA substrate containing DHU, although the cleavage efficiency turned out to be much slower compared to in a previous work [51] (the product accumulation level was ~20% after 30 min). This discrepancy could be due to the differences in the reaction conditions and to the choice of the substrates. Although EndoQ was found to exhibit cleavage activity toward some other NIR substrates such as 5hU and 5hC [22,25,51], it turned out to be inactive toward αA- and εA-containing DNA. EndoQ also showed no activity toward the exo-substrate.

**Figure 6:**
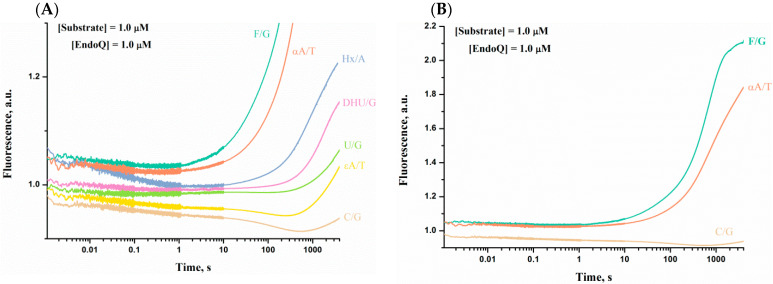
Interactions of EndoQ with substrate F/G, αA/T, Hx/A, DHU/G, U/G, or εA/T or undamaged DNA (**A**). A comparison of the kinetic traces characterizing the interaction of the enzyme with substrates F/G, αA/T, or undamaged DNA (**B**). [Enzyme] = [DNA substrate] = 1.0 μM.

To elucidate whether EndoQ was still able to bind the DNA substrates in question, fluorescence curves reflecting the interactions of the enzyme with a damaged DNA substrate containing αA, εA, or DHU or with the undamaged DNA substrate were recorded by the stopped-flow kinetic method (Figure 6). All time courses were recorded within time intervals of up to 4000 s; the fluorescence curves for the interactions of the EndoQ with DNA substrate F/G, Hx/A, or U/G are presented in the graph for comparison. The changes in the FRET signal in the case of DNA substrates αA/T, εA/T, and C/G most likely correspond only to the binding by EndoQ and subsequent conformational transformation of the enzyme–substrate complexes because it was demonstrated by PAGE that no significant cleavage of these substrates occurred within the given time interval. The cleavage of substrate DHU/G took place during the period of recording, although it was significantly slower than that of F-, Hx- or U-containing DNA.

The fluorescent trace recorded for interactions between EndoQ and DNA substrate DHU/G consisted of the initial two-phase decrease in the FRET signal up to 1 s followed by a slow increase that transitioned to a fast intensive increase in the signal; this increase tended to slow down only at 4000 s. The character of the changes was very much reminiscent of the kinetic curves from the interaction of EndoQ with Hx/A and U/G. Of note, the stage of the intensive increase in the signal of the fluorescent traces started first in the case of Hx/A, followed by DNA substrate DHU/G and then by U/G. If this phase corresponds to the cleavage process exclusively, then the order (according to the PAGE experiments) should be as follows: U/G, Hx/A, and DHU/G.

Another example indicating that the intensive growth of the FRET signal phase corresponds to several complicated processes (including the cleavage process) is the fluorescence time course of the interaction of EndoQ with αA-containing DNA substrates (Figure 6B). This experiment showed an initial decrease in the FRET signal with small amplitude, followed by an increase in the signal that started even earlier relative to Hx/A or DHU/G. This phase could not represent the catalytic cleavage of the substrate because the PAGE experiments showed no cleavage product during the time interval under study (Figure 5).

For the EndoQ interaction with the DNA substrate containing εA and with the undamaged DNA, the profile of the kinetic curves was quite similar between these substrates and different from profiles of the other substrates. It contained an additional phase of a decrease in the signal following the initial decrease phase, and this pattern seemed to be common among all the tested substrates. Notably, this phase was followed by an increase in fluorescence after 250 s in the case of substrate εA/T and after 500 s for G/C. This increase had a much smaller amplitude than in the case of F/G and αA/T but was comparable to that of U/G.

It has been reported that in addition to Hx and U (considered to be the primary substrates for EndoQ), this enzyme is also able to cleave DNA containing an AP site [48] and some lesions chemically close to Hx and U, namely, DHU, 5hU, and 5hC [51]. Those authors have proposed that any other lesion substantially different from those discussed above would not be accommodated by the active site of the enzyme. The failure of EndoQ to cleave such lesions as εA and αA supports this theory; also, it is possible that the DNA containing these lesions can still be bound by EndoQ.

## 3. Discussion

Hyperthermophilic archaea survive under very aggressive environmental conditions by occupying niches inaccessible to representatives of other domains of life. One of the most studied members of hyperthermophilic archaea is *P. furiosus*, and it grows optimally at temperatures approaching 100 °C [56]. The ability to survive such severe living conditions must be based on extraordinarily efficient DNA repair mechanisms. Recent research led to the discovery of a novel endonuclease from *P. furiosus:* EndoQ [48]. Homologs of this enzyme that participate in the repair of such DNA lesions as U, Hx, and AP site, and some others [51] have been found in several orders of microbes from Archaea and in some members of the Bacteria domain, but no such representatives of Eukarya have been found to date [49].

Our results support the idea that the activity of EndoQ depends on the presence of Mg^2+^ [48]. It is known that three Zn^2+^ cations are located in the structure of EndoQ, and amino acid residues have been proposed to form the Mg^2+^-binding site in a closed DNA-bound conformation of the enzyme [50]. In our experiments, we observed a complete loss of the catalytic activity in EndoQ after the treatment of the enzyme with EDTA, and the activity was restored by the addition of only Mg^2+^. This observation led to a supposition that apparently no other divalent cation can effectively take the place of Mg^2+^ in its binding center.

It is noteworthy that EndoQ turned out to be more catalytically active on ssDNA containing U and Hx but showed almost no activity toward F-site in ssDNA in our work; also, EndoQ was most active toward an abasic site in dsDNA, in agreement with data obtained earlier [48,51].

It should be noted that among representative AP endonucleases and DNA glycosylases the ability to cleave single-stranded damaged DNA is significantly varied. For example, in *E. coli*, the hypoxanthine DNA glycosylase function is performed by AlkA, also known as 3-methyladenine DNA glycosylase [57]. Homologous enzymes of AlkA in human (AAG), rat (APDG) and yeast (MAG) also exhibit hypoxanthine activity; however, none of these enzymes cleave ssDNA containing hypoxanthine [57]. Human AP endonuclease APE1 is capable of cleaving AP sites in ssDNA, but its processivity is approximately 20 times lower in such regions compared to dsDNA [58]. Finally, DNA glycosylase NEIL1 removes DNA damage such as 5,6-dihydroxyuracil, 5-hydroxyuracil, and others, and this enzyme has specificity for lesions located in incomplete dsDNA and is active toward ssDNA [59,60]. Uracil DNA glycosylases are also capable of removing uracil from ssDNA [61]. It could be assumed that the ability of EndoQ to cleave ssDNA could have important biological function for repairing partially melted DNA regions caused by high temperatures of thermophilic archaea habitats. Moreover, data obtained on the ability of EndoQ to efficiently cleave Hx and U, but not F-site, in ssDNA suggest that one of the factors of substrate recognition is the formation of direct contacts with the damaged base inside the active site. Structural evidence suggests that most of the contacts that EndoQ forms with DNA involve the damaged strand on the 3′ and 5′ sides of the lesion [50]. The efficient cleavage of U and Hx in ssDNA is consistent with these findings, but inefficient cleavage of F-site, DHU, and 5hU in a single-stranded context [51] suggests that there must be some cardinal difference between these substrates that becomes well pronounced when there is no complementary strand. Structural data suggest that near the active site, EndoQ possesses an additional recognition pocket that is able to enter several hydrogen bonds with U and Hx specifically [50,53]. It has been suggested that this pocket implements the positive selection of a lesion along with negative selection via steric hindrance, preventing undamaged nucleotides and other lesions (with the exception of close chemical analogs of Hx and U [51]) from being accommodated in the active site [50]. The absence of the nucleobase moiety in F-site along with its destabilizing effect on DNA structure [62] makes it an even more suitable substrate than Hx or U, as evidenced by a biochemical analysis conducted earlier [51] and by our results. Nevertheless, it is possible that the hydrogen bonds between the recognition pocket and U/Hx bases contribute to the stability of the EndoQ–DNA complex. This stabilization apparently does not fully compensate for the energy costs for the flipping out and the accommodation of the damaged base in the active site of the enzyme, but this stabilization becomes crucial when it comes to the binding of ssDNA.

The substrate specificity mechanism of EndoQ could be similar to the well-studied AP endonucleases from the Xth and Nfo families as well as multiple DNA glycosylases, which also recognize in DNA a single-nucleotide lesion. It was revealed that DNA glycosylases from six structural families have different structures of the DNA-binding site and of the catalytically active site and distinct functional amino acid residues involved in specific recognition of a damaged nucleotide and in catalysis, but unexpectedly, they have common features of interaction with damaged DNA [63,64,65,66,67,68,69,70]. Indeed, almost all DNA glycosylases bend DNA and flip out the damaged nucleotide from the DNA double helix into the active-site pocket, where its final verification takes place. This strategy of damage recognition is also suitable for AP endonucleases from both well-known Xth and Nfo structural families [34,38,40,41,42,71,72,73,74]. Shiraishi and Iwai’s model of the mechanism behind the catalytic activity of EndoQ implies the flipping of the damaged base into the recognition pocket of the enzyme and subsequent discrimination of the lesion on the basis of affinity for the recognition site. It has been proposed that in the case of the optimal accommodation of the base in the active site, the flipped-out conformation is then stabilized by the insertion of an amino acid residue of the enzyme into the DNA duplex in place of the flipped-out base, and this event could trigger the cleavage process, but this idea has not found confirmation in further structural studies [50,51]. Similarly to various DNA repair enzymes, EndoQ’s endonuclease activity is facilitated by the flipping of a damaged nucleotide into an extrahelical position [50]. Nevertheless, unlike AP endonucleases from structural families Xth and Nfo and unlike DNA glycosylases [33,75,76], EndoQ does not insert amino acid residues into the base stack that stabilizes DNA in the flipped-out conformation. Instead, base flipping is promoted by a variety of contacts of amino acid residues with the DNA backbone of unstacked base pairs neighboring the damaged nucleotide [50]. To sum up, we can state that the step-by-step catalytic mechanism of the reaction between EndoQ and a DNA substrate remains unclear. It is most likely that the formation of the contacts with DNA, mainly with the damaged strand, either precedes the flipping-out stage or occurs simultaneously. Then, the hydrogen bonds in the Hx/U-recognizing pocket are formed, and the enzyme–substrate complex becomes catalytically competent.

According to recent structural research, DNA bound to EndoQ adopts a highly distorted conformation. It has also been demonstrated in several experiments, including ours, that instability of the DNA double helix in the damage area is an important factor for the substrate recognition and cleavage efficiency of EndoQ [50,51]. Our examination of fluorescence time courses representing conformational changes of the dye-labeled DNA substrates during interaction with EndoQ supports the hypothesis that DNA is substantially bent during these interactions. The initial decrease in the FRET signal is present in kinetic traces obtained for all of the tested lesions. This decrease is due to the FAM and BHQ1 coming closer in the DNA duplex bent by EndoQ. This process starts at early stages of the fluorescence recording, meaning that it probably corresponds to the formation of the initial nonspecific enzyme–DNA complex. This notion is consistent with the finding that a similar decrease phase was found in the fluorescent traces registered for such uncleavable DNA substrates as undamaged C/G and εA.

Of note, for some U-containing DNA substrates (namely, U/A), there was an additional well-pronounced stage characterized by a subsequent increase and decrease in the FRET signal. This observation indicates that the DNA duplex containing the U/A base pair undergoes additional changes in the distance between FAM and BHQ1, as if the DNA were relaxed and then bent again during adjustments in the enzyme–substrate complex. It is possible that a similar process takes place with the other substrates as well, but the changes are less pronounced. The finding that this process yields the profile with the most striking changes in the case of DNA substrate U/A could be explained by the stability of this particular base pair.

Overall, our data from the stopped-flow experiments indicate that the base-pairing effects contribute to the formation of the enzyme–substrate complex and to the catalytic activity of EndoQ.

The differences among DNA substrates Hx/A, U/A, and F/G in the profile of the fluorescence traces characterizing the binding and cleavage by EndoQ suggest that the enzyme’s interactions with these substrates are not identical. The assembly of the enzyme–substrate complex apparently goes through slightly different pathways, resulting in different types of alterations in the DNA conformations. The results of the stopped-flow experiments along with the PAGE data indicate that U is cleaved more effectively than Hx, and this finding could be a consequence of the more efficient formation of the complex with U. These data are in agreement with earlier reports and could be due to a difference in the kinetics of the flipping between U and Hx bases [53].

**Figure 7:**
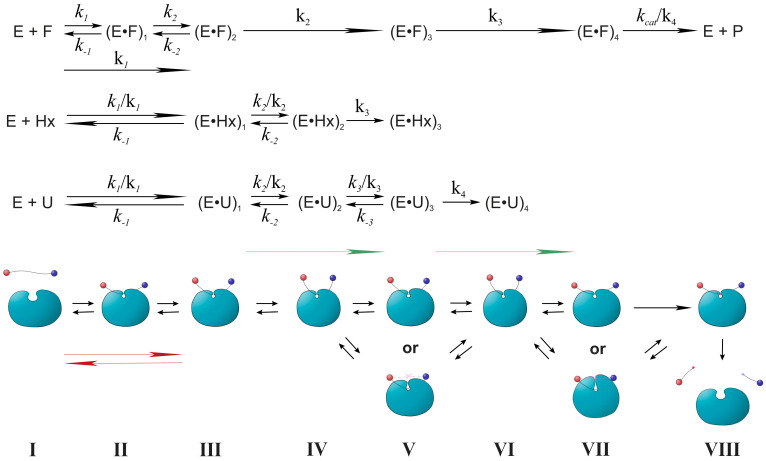
The general scheme of interactions between the EndoQ enzyme and DNA substrates containing an F-site, Hx, or U as damage. E is the enzyme; F, Hx, and U are DNA substrates containing an F-site, Hx, or U as a lesion, respectively; (E·F)_i_, (E·Hx)_i_, and (E·U)_i_ denote enzyme–substrate complexes of EndoQ with F-, Hx-, or U-containing DNA, respectively; P represents the product of the catalytic cleavage; *k*_i_ stands for the observed rate constants; *k_i_* is forward and reverse rate constants; *k_cat_* is the catalytic rate constant. Green arrows are the pathway registered only in the case of the F-site-containing substrate. Red arrows: the pathway documented only for Hx-containing and U-containing substrates.

After the comparison of our findings with the available literature data, we propose a step-by-step catalytic mechanism of the reaction between the EndoQ enzyme and F-site-, Hx-, or U-containing DNA (Figure 7). The first step, matching the initial binding of the DNA substrate by the enzyme and formation of complex **III**, is accompanied by the bending of DNA. This bending leads to a decrease in the FRET signal. This stage seems to be a common feature of the interaction of EndoQ with each of the three DNA substrates. Moreover, it is likely to be universal among all our tested substrates, including the uncleavable ones. Thus, we associate this first step with the nonspecific binding to the DNA substrate and attempts of the nucleotide to evert into the active site of the enzyme. Notably, this stage was found to consist of two phases of subsequent bending of the DNA in the case of the F-site. The second step of this mechanism reflects the transformation of complex **III** into complex **IV** (characterized by further bending of the DNA substrate) and turned out to be specific for the Hx- and U-containing DNA substrates. For the F-site, the formation of enzyme–substrate complex **IV** was not detectable. This finding led us to the supposition that this step could represent the process of the accommodation of an Hx or U base in the specific recognition pocket and the formation of hydrogen bonds with the damaged base. The third step of the kinetic mechanism is accompanied by a small increase in the FRET signal. This could mean that the bent conformation of the DNA substrate in the complex with the enzyme slightly relaxes during subsequent conformational tuning in the active site, thereby causing an increase in distance between FAM and BHQ1. Another possibility is that during the formation of complex **V**, parts of the enzyme change their positioning relative to the DNA, thus leading to partial shielding of FAM from BHQ1. The next step, denoting transformation of complex **V** into complex **VI**, was registered only in the case of U-containing DNA. Formation of this complex, accompanied by further bending of the DNA, may reflect differences in the accommodation of Hx and U by the active site of the enzyme. On the contrary, for the F-site-containing DNA, we noticed another step in the relaxation of the bent DNA or further shielding of the FAM from BHQ1 by parts of the EndoQ protein. The increase in the FRET signal with considerable amplitude at this step is more or less appreciable in the kinetic curves characterizing the uncleavable DNA substrates, meaning that this process may represent some nonspecific interactions of the EndoQ enzyme with DNA at later time points. Finally, the step of catalytic cleavage of the DNA substrate and of dissociation of the enzyme–product complex (state **VIII**) was registered only for the F-site-containing DNA. It is possible that our failure to detect and characterize this process with Hx- and U-containing substrates is explained not only by the slower cleavage of these substrates but also by a finding that after the cleavage, the products remain bound to the enzyme [47].

In summary, this work allows us to outline a model of recognition of different substrates by *P. furiosus* EndoQ and provides some insights into the process of formation of a substrate–enzyme complex with Hx-, U-, or F-site-containing DNA. The obtained data first of all indicate that the interaction of EndoQ with DNA containing the Hx, U, or F-site as damage is a complex multi-stage process, the various stages of which differ significantly for different damages and different nucleotides opposite the damage. However, in order to accurately address the observed individual stages with the molecular events occurring in the enzyme–substrate complex, further studies should be performed. As such, the creation of mutant forms of the enzyme in which certain binding steps or catalytic activity are impaired, as was carried out in our previous study of human DNA glycosylase OGG1 [77], would allow a better understanding of the different reaction stages. In addition, Mg^2+^-induced enzyme activation in the preformed enzyme–substrate complex would help distinguish between the complex formation and the catalytic reaction stages [78]. Also, the analysis of changes occurring with the enzyme or substrate conformation during their interaction by recording changes in the fluorescence intensity of various fluorophores, as reported in [79], would make a significant contribution to confirming or revising the scheme of interaction of EndoQ with various substrates that we have proposed.

## 4. Materials and Methods

### 4.1. Oligonucleotides

The synthesis of the oligonucleotides (Table 5) was carried out on an ASM-800DNA/RNA synthesizer (Biosset, Novosibirsk, Russia) by means of standard commercial phosphoramidites and CPG solid supports from Glen Research (Sterling, VA, USA). The oligonucleotides were deprotected according to the manufacturer’s protocols and were purified by high-performance liquid chromatography. Oligonucleotide homogeneity was checked by denaturing 20% polyacrylamide gel electrophoresis (PAGE). Concentrations of oligonucleotides were calculated from their absorbance at 260 nm (A_260_). Oligonucleotide duplexes were prepared by annealing oligonucleotide strands at a 1:1 molar ratio.

### 4.2. Enzyme Purification

**Figure 8:**
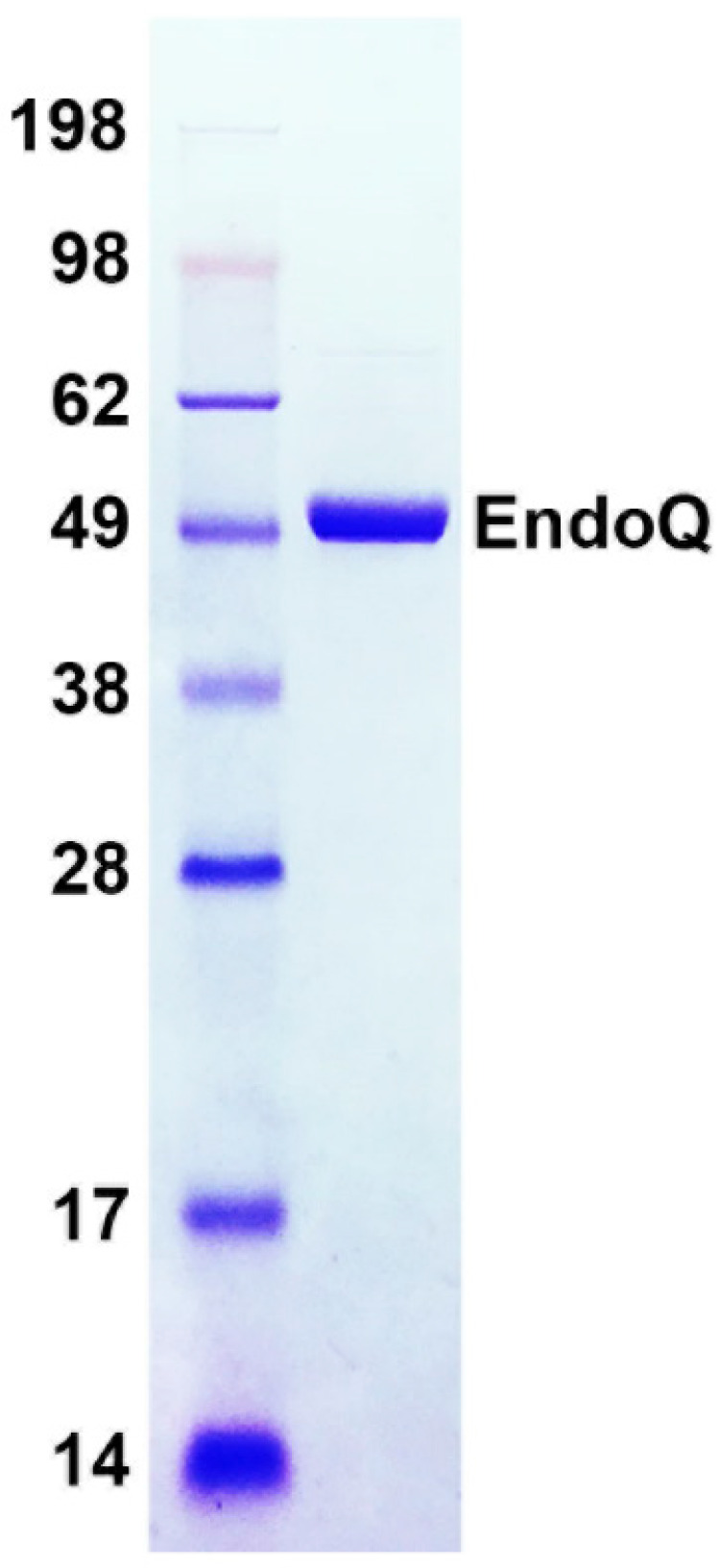
Analysis of the EndoQ enzyme purity by PAGE. Coomassie Brilliant Blue R-250 (Sangon Biotech, Shanghai, China) staining was performed for protein visualization.

The EndoQ enzyme from *P. furiosus* was isolated from *E. coli* ArcticExpress (DE3) cells transformed with plasmid pET28c carrying a relevant N-terminal His-tagged gene construct. To purify the enzyme expressed as recombinant proteins, 2 L of culture (in YT broth) of *E. coli* cells carrying the encoding vector construct was grown with 50 µg/mL kanamycin, 20 µg/mL gentamycin, and 10 µg/mL tetracycline at 37 °C until A_600_ reached 0.6–0.7; the expression of the enzyme was induced during 24 h at 16 °C with 0.2 mM isopropyl β-D-1-thiogalactopyranoside. The cells were harvested by centrifugation (5000× *g*, 20 min) and then resuspended in a buffer (20 mM HEPES-KOH pH 7.8, 40 mM NaCl), followed by cell lysis by means of a French press. All the purification procedures were carried out at 4 °C. The homogenate was centrifuged at 40,000× *g* for 45 min, NaCl concentration in the supernatant was brought to 400 mM, and the supernatant was passed through a column packed with 30 mL of Q-Sepharose Fast Flow (Cytiva, GE Healthcare Life Sciences, Cicago, IL, USA) pre-equilibrated in the same buffer. Flow-through fractions containing an enzyme were pooled, supplemented with 20 mM imidazole, and loaded on a 1 mL HiTrap-Chelating^TM^ column (Cytiva, GE Healthcare Life Sciences, Cicago, IL, USA). The bound protein was eluted with a linear 20 → 600 mM gradient of imidazole. The fraction containing the EndoQ protein was dialyzed against a buffer (20 mM Tris-HCl pH 7.5, 200 mM NaCl, 5 mM DTT, and 20% of glycerol) and stored at −20 °C. The homogeneity of the protein was verified by SDS-PAGE (Figure 8); the protein concentration was measured by the Bradford method.

### 4.3. Endonuclease Assay

6-Carboxyfluorescein (FAM)-5′-labeled oligonucleotides were subjected to experiments on the separation of cleavage products by PAGE. The dependence of the activity of EndoQ on temperature was reported earlier [47]. According to the obtained data, enzyme activity at 75 °C was dramatically greater than at 60 °C and 40 °C. Nevertheless, due to temperature limitations of the stopped-flow fluorescent instrument, all experiments in the present study were performed at 40 °C. AP endonuclease assays with all DNA substrates were performed at 40 °C, with the substrate and the enzyme being kept at 40 °C for at least 4 min before the reaction, in 10 µL reactions containing reaction buffer consisting of 50 mM Tris-HCl (pH 8.0), 50 mM KCl, 2 mM MgCl_2_, 1 mM dithiothreitol, and 7% glycerol (*v*/*v*). The substrate concentration chosen for comparing the activity of the enzyme with different substrates was 1.0 µM, and the concentration of the enzyme was 1.0 µM as well. The reaction was initiated by the addition of the enzyme. Aliquots of the reaction mixture were withdrawn, immediately quenched with 10 mL of a gel-loading dye containing 7 M urea and 25 mM EDTA, followed by heating at 95 °C for 3 min, and were loaded on a 20% (*w*/*v*) polyacrylamide/7 M urea gel. PAGE (gel concentration: 20%) was performed under denaturing conditions (7 M urea) at 55 °C and a voltage of 200–300 V. The gels were visualized using an E-Box CX.5TS gel-documenting system (Vilber Lourmat, Marne-la-Vallée, France), and the bands were quantified by scanning densitometry in the Gel-Pro Analyzer software, v.4.0 (Media Cybernetics, Rockville, MD, USA). In some cases, to visualize in figures trace amounts of cleavage products, it was required to increase the sensitivity of the gel documentation system, which resulted in light and shade effects. This effect did not affect the efficacy of DNA cleavage or interpretation of the results obtained.

### 4.4. Assays of the Impact of Divalent Cations

To study the effect of different divalent cations on the endonuclease activity of EndoQ toward DNA substrate “F/G” (Table 1), the enzyme was initially incubated with 1.0 mM EDTA (15 min) on ice to chelate any divalent metal ions. Then, the enzyme was incubated (10 min) with a certain concentration (0, 0.05, 0.1, 0.5, 1.0, 1.5, 2.0, 2.5, 3.0, or 5.0 mM) of MeCl_2_ (where Me is Mg, Mn, Ca, or Zn). Cleavage of the F/G substrate was initiated by the addition of EndoQ. Aliquots of the reaction mixture were taken at 3 min (for the Mg^2+^-containing solutions) or 30 min for the other divalent cations and immediately quenched as described above.

### 4.5. Fluorescence Stopped-Flow Experiments

Pre-steady-state kinetics were analyzed by the stopped-flow technique using an SX.18 stopped-flow spectrometer (Applied Photophysics Ltd., Leatherhead, UK). The fluorescence of FAM was excited at 494 nm, and the Förster resonance energy transfer (FRET) signal was monitored at wavelengths ≥530 nm by means of an OG-530 filter (Schott, Mainz, Germany). The dead time of the instrument is 1.4 ms. Typically, each trace shown is an average of three or more individual experiments. Experimental error was less than 5%. All the experiments were conducted in reaction buffer consisting of 50 mM Tris-HCl (pH 8), 50 mM KCl, and 1 mM MgCl_2_. The solutions containing the enzyme and substrate were loaded into two separate syringes of the stopped-flow instrument and were incubated for an additional 4 min at 40 °C prior to mixing. The reported concentrations of reactants are those in the reaction chamber after the mixing.

### 4.6. Analysis of Kinetic Data

The sets of kinetic curves obtained for the interactions of DNA substrates F/N, Hx/N, or U/N (where N = A, C, G, or T) with EndoQ were fitted to the following exponential equation with amplitudes A_i_ and first-order rate constants *k*_obsi_ (with the number of exponents *n* being 2 to 4 depending on the curve) using the Origin 10.1 software (Originlab Corp., Northampton, MA, USA):

(1) $$y=\sum_{i=1}^{n}A_{i}\exp\left({-k}_{obsi}t\right)+offset$$

The sets of kinetic curves obtained at different concentrations of DNA substrates F/G, Hx/A, and U/A during interactions with EndoQ were analyzed in the DynaFit 4.0 software (BioKin, Pullman, WA, USA) [80], as described elsewhere [69,81,82,83].

The kinetic curves represent changes in the FRET signal in the course of the reaction owing to sequential formation and subsequent transformation of the DNA–enzyme complex and its conformers. The stopped-flow fluorescence traces were directly fitted to fluorescence intensity (*F*) at any reaction time point (t) as the sum of the background fluorescence and fluorescence intensity values of each intermediate complex formed by the enzyme with DNA:

(2) $$\;F=F_{b}\sum_{i=0}^{n}f_{i}\times {\left[ES\right]}_{i}$$

where *F_b_* is the background fluorescence or an equipment-related photomultiplier parameter (“noise”), and *f_i_* is the molar response coefficient of the *i*th intermediate ES*_i_* (*i* = 0 corresponds to the free protein and *i* > 0 to the enzyme–DNA complexes).

Concentrations of each species in the mechanisms are described by a set of differential equations according to a kinetic scheme (see the Results section). The software performs numerical integration of a system of ordinary differential equations with subsequent nonlinear least-squares regression analysis. In the fits, the values of all relevant rate constants for the forward and reverse reactions are optimized, as are the specific molar “response factors” for all intermediate complexes.

## Figures and Tables

**Table 1:** Rate constants of the interaction of EndoQ with F-containing DNA substrates. The F-site was placed opposite A, C, G, or T. The constants were obtained via fitting of the individual FRET traces to several exponential equations.

	F/A	F/C	F/G	F/T	Signal
*k*_1_, s^−1^	33 ± 1	-	34 ± 4	53 ± 4	Initial decrease
*k*_2_, s^−1^	-	2.15 ± 0.06	-	-	Slow increase
*k*_3_, s^−1^	0.030 ± 0.001	-	-	-	Beginning of final increase
*k*_4_, s^−1^	0.005 ± 0.001	0.004 ± 0.001	0.003 ± 0.001	0.004 ± 0.001	Final increase

**Table 2:** Constants of the interaction of EndoQ with Hx-containing DNA substrates. Damaged base was paired with A, C, G, or T. Constants were obtained through fitting of the individual FRET traces to several exponential equations.

	Hx/A	Hx/C	Hx/G	Hx/T	Signal
*k*_1_, s^−1^	50 ± 20	60 ± 30	70 ± 30	50 ± 20	Initial decrease (1st phase)
*k*_2_, s^−1^	5 ± 2	5 ± 2	8 ± 5	5 ± 4	Initial decrease (2nd phase)
*k*_3_, s^−1^	-	-	0.07 ± 0.04	0.12 ± 0.08	Slow increase

f the data are not presented as mean ± standard deviation, then the parameter in question was fixed during the fitting.

**Table 3:** Rate constants of the interaction of EndoQ with U-containing DNA substrates. The damaged base was paired with A, C, G, or T. The constants were obtained via fitting of the individual FRET traces to several exponential equations.

	U/A	U/C	U/G	U/T	Signal
*k*_1_, s^−1^	36 ± 6	36	60 ± 30	60 ± 20	Initial decrease (1st phase)
*k*_2_, s^−1^	3.5 ± 0.9	2.0	-	-	Initial decrease (2nd phase)
*k*_3_, s^−1^	0.13 ± 0.03	0.9	0.3 ± 0.2	0.3 ± 0.1	Following increase phase
*k*_4_, s^−1^	0.005	0.038	0.03 ± 0.1	0.013 ± 0.009	Second decrease stage

If the data are not presented as mean ± standard deviation, then the parameter in question was fixed during the fitting.

**Table 4:** Rate and equilibrium constants of the interaction of EndoQ with DNA substrate F/G, Hx/A, or U/A, as determined by nonlinear least-squares regression analysis of the FRET traces.

Constant	F/G	Hx/A	U/A
*k*_1_ × 10^−6^, M^−1^s^−1^	1.4 ± 0.5	17 ± 3	74 ± 7
*k_-_*_1_, s^−1^	26 ± 7	99 ± 7	100 ± 20
*K*_1_ × 10^−6^, M^−1^	0.05 ± 0.03	0.17 ± 0.04	0.8 ± 0.2
*k*_2_, s^−1^	0.17 ± 0.04	0.8 ± 0.2	3.9 ± 0.5
*k_-_*_2_, s^−1^	0.032 ± 0.005	6.2 ± 0.2	8.0 ± 0.3
*K*_2_	5 ± 2	0.13 ± 0.04	0.50 ± 0.08
*k*_3_, s^−1^			0.10 ± 0.02
*k_-_*_3_, s^−1^			0.096 ± 0.004
*K*_3_			1.0 ± 0.3
*k_cat_*, s^−1^	0.008 ± 0.001		

**Table 5:** Sequence of oligonucleotides.

Shorthand	Sequence
X/N X = F-site, Hx, U N = A, C, G, T	5′ - **FAM**-GCTCA**X**GTACAGAGCTG - 3′ 3′ - CGAGT**N**CATGTCTCGAC-**BHQ1** - 5′
Y/T Y = αA, εA	5′ - **FAM**-GCTCA**Y**GTACAGAGCTG - 3′ 3′ - CGAGT**T**CATGTCTCGAC-**BHQ1** - 5′
Z/G Y = DHU, C	5′ - **FAM**-GCTCA**Z**GTACAGAGCTG - 3′ 3′ - CGAGT**G**CATGTCTCGAC-**BHQ1** - 5′
Exo-substrate	5′ - GTGTCACCACTGCTCACGTACAGAGCTG - 3′ 3′ - CGAGTGCATGTCTCGAC- **FAM** - 5′

## Data Availability

Data are available from N.A.K. upon request. Tel.: +7-(383)-363-5175, e-mail: nikita.kuznetsov@niboch.nsc.ru.
